# A Transcriptomic Analysis of Tobacco Leaf with the Functional Loss of the Plastid *rpoB* Operon Caused by TALEN-Mediated Double-Strand Breakage

**DOI:** 10.3390/plants11212860

**Published:** 2022-10-26

**Authors:** Yu-Chang Liu, Chih-Hao Huang, Ching-Chun Chang

**Affiliations:** 1 Institute of Biotechnology, National Cheng Kung University, Tainan 701, Taiwan; 2 Department of Biotechnology and Bioindustry Sciences, National Cheng Kung University, Tainan 701, Taiwan; 3 Institute of Tropical Plant Sciences and Microbiology, National Cheng Kung University, Tainan 701, Taiwan

**Keywords:** plastid, nucleus-encoded polymerase, plastid-encoded polymerase, transcription activator-like effector nuclease, RNA-Seq

## Abstract

At least two sets of RNA polymerase (RNAP), nucleus (NEP)- and plastid (PEP)-encoded polymerases, recognizing distinct promoters exist in the plastids of land plants. Most plastid genes are regulated by multiple promoters with different strengths in their response to developmental stages and environmental cues. Recently, we applied chloroplast-targeted transcription activator-like effector nuclease (cpTALEN) technology to site-specifically cause double-strand DNA breaks in the *rpoB* gene of tobacco, which encodes the β-subunit of PEP. The repair of damaged chloroplast DNA (cpDNA) through microhomology-mediated recombination caused the functional loss of the *rpoB* operon and resulted in the heterotrophic growth of an albino plant. We conducted a genome-wide analysis of the steady state of gene expression in the leaf tissue of PEP-deficient tobacco by RNA-Seq and compared it with that of wild-type plants. The expression of NEP genes was up-regulated in PEP-deficient tobacco; in particular, the level of *RpoT3* transcripts encoding the specifically plastid-targeted NEP was significantly increased. Alongside most housekeeping genes, NEP also plays an important role in the regulation of gene expression involved in photosynthesis. In contrast, alongside the photosynthesis-related genes, PEP also plays an important role in the regulation of gene expression involved in some housekeeping functions. Furthermore, the mitochondrial DNA copy number and the level of most mitochondrial protein-coding transcripts were slightly increased in PEP-deficient tobacco. The disruption of PEP function not only affected plastid gene expression, but also nuclear and mitochondrial gene expression. This study demonstrated the intercompartmental retrograde signaling in the regulation of gene expression.

## 1. Introduction

Plant chloroplasts are derived from cyanobacteria through endosymbiotic evolution. The chloroplast DNA (cpDNA) of higher plants generally ranges from 120 to 160 kb, and usually encodes 110 to 120 genes with high conservation, which consists of rRNA, tRNA and protein-coding genes mostly involved in transcription, translation and photosynthesis [1,2]. The process of plastid transcription is complicated in land plants, with at least two sets of RNA polymerase (RNAP) [3,4]. One is plastid-encoded RNA polymerase (PEP), which is *E. coli*-like, and contains the α, β, β’ and β” subunits encoded by the *rpoA*, *rpoB*, *rpoC1* and *rpoC2* genes, respectively. The specific activity of the core PEP is regulated by up to six nucleus-encoded σ-like transcription initiation factors in *Arabidopsis* [5], which are responsible for recognizing *E. coli*-like “−10 and −35” promoters. The other type is the nucleus-encoded RNA polymerase (NEP), which is a bacteriophage T3/T7-like single peptide that can recognize a loose YRTA promoter [4,6]. Monocotyledonous plants (e.g., maize and wheat) possess two NEPs with one specifically targeting the mitochondria and another specifically targeting the chloroplasts [3]. In dicots (e.g., tobacco and *Arabidopsis*), the nuclear genome harbors three different sets of NEP: *rpoT1* or *rpoTm*, *rpoT2* or *rpoTmp* and *rpoT3* or *rpoTp* [7,8,9,10]. RpoT1 (RpoTm) and RpoT3 (RpoTp) are exclusively targeted to the mitochondria and chloroplasts, respectively, and RpoT2 (RpoTmp) is dual targeted to the mitochondria and chloroplasts [7]. The two phage-type RNA polymerases (RpoTp and RpoTmp) have overlapping as well as gene-specific functions in the transcription of plastid genes in *Arabidopsis* [10,11]. In *Arabidopsis*, a T-DNA insertion mutant of the *rpoTp* gene resulted in a significant reduction in the size, morphology, and number of chloroplasts and thus severely impaired plant growth and reduced pigmentation [11]. In contrast, overexpression of the RpoTp gene in transgenic tobacco plants could enhance the transcription from certain NEP promoters [8].

Further complicating transcription, multiple promoters are commonly present in chloroplast genes for regulating gene expression by adjusting the number of active promoters and/or altering promoter strength at a distinct developmental stage or in response to an environmental cue [4]. For example, five promoters with four promoters containing *E. coli*-like “−10 and −35” consensus sequence motifs exist in the *atpB* operon in tobacco [12]. Previously, deletion of the *rpoA, rpoB*, *rpoC1* or *rpoC2* encoding subunits of PEP by plastid transformation resulted in photosynthetic incompetence in tobacco, but the plants could still heterotrophically grow in a medium with a carbon source [13,14,15]. An initial analysis of PEP-deficient mutants compared with wild-type tobacco suggested that PEP is mainly responsible for transcribing the photosynthesis-related genes (designated as Class I genes) [6,16], and NEP is mainly responsible for transcribing all the essential housekeeping genes (Class III genes) [16]. For instance, transcription of the *rpoB* operon, consisting of *rpoB*, *rpoC1* and *rpoC2*, is exclusively carried out by a highly regulated NEP promoter in tobacco [4,17]. The genes that have functional promoters for both NEP and PEP polymerases are designated as Class II genes, such as *atpB* and *clpP* [6,8]. However, more extensive analysis of plastid gene expression in PEP-deficient tobacco mutants and the barley albostrians mutant demonstrated that NEP could transcribe all parts of cpDNA, though the gene expression profiles were distinct between PEP-deficient plant and the wild-type [6,18,19]. In addition, in barley, most plastid genes (including photosynthesis-related genes) have both PEP and NEP promoters [19], which indicates that functional integration of PEP and NEP into the regulation of gene expression is complex.

Transcription activator-like effector nucleases (TALENs) play an important role in genome editing because of the high specificity, the feasibility of organellar targeting and their high efficiency in heterochromatin regions [20,21,22,23,24,25]. For instance, the use of TALENs fused with mitochondria localization signals in the N-terminus (mitoTALENs) could successfully restore male fertility in rice and rapeseed CMS varieties by knocking out cytoplasmic male sterility (CMS)-associated genes [21]. Bacterial cytidine deaminase fused to the DNA binding domains of TALENs and chloroplast transit peptide could homoplasmically substitute the targeted Cs to Ts in the plastid DNA of *Arabidopsis,* and the targeted base editing mutations could be inherited by their offspring [24]. Recently, we used a leaf-specific *rbcS* promoter to drive the expression of TALENs fused with a transit peptide to site-specifically caused double-strand DNA breaks in the *rpoB* gene of tobacco [25]. Both photosynthetically competent and incompetent transgenic tobacco lines were generated [25]. In an albino line, the repair of damaged cpDNA through microhomology-mediated recombination caused the functional loss of the *rpoB* operon and resulted in the heterotrophic growth of the plant [25]. In this study, we conducted a genome-wide analysis of the gene expression in the leaf tissue between PEP-deficient tobacco and wild-type plants by RNA-Seq. Our results suggested that alongside most housekeeping genes, NEPs also play an important role in the regulation of genes involved in the photosynthetic apparatus. Moreover, along with the photosynthesis-related genes, PEP also plays an important role in the regulation of some genes involved in housekeeping functions. In addition, the expression of NEP (*RpoT1*, *2* and *3*) genes, particularly *RpoT3*, was significantly up-regulated in albino plants with a functional loss of the *rpoB* operon. Furthermore, the mitochondrial DNA (mtDNA) copy numbers as well as gene expression level were slightly increased in PEP-deficient tobacco. Our study strongly demonstrated intercompartmental retrograde signaling in the regulation of gene expression.

## 2. Methods

### 2.1. Plant Materials 

Transgenic tobacco lines with nuclear integration of recombinant TALENs genes [25] and untransformed tobacco (*Nicotiana tabacum* cv. Petit Havana) were grown in half-strength MS media at 26 °C at a 16/8 h light−dark cycle in a growth chamber. 

### 2.2. DNA Extraction and qPCR 

Total DNA was isolated from the leaves of tobacco seedlings as described [3]. qPCR analysis was used to investigate the relative number of mitochondrial gene copies in plants. The qPCR reactions were performed with a 20 μL volume containing 10 μL GoTaq qPCR Master Mix (Promega, Madison, WI, USA), 100 ng DNA as a template and the indicated primers (Supplementary Table S1) at a final concentration of 100 nM by using the CFX connect Real-Time System (BioRad, Hercules, CA, USA) for 2 min at 95 °C and 40–45 cycles of 15 s at 95 °C and 60 s at 60 °C. The qPCR assays were conducted with the biological replicates and for each with triplicate reaction.

### 2.3. RNA Extraction and qRT-PCR

Total RNA was isolated from the leaf tissues of tobacco plants at the indicated developmental stages by using the Tri-plant RNA isolation reagent (Geneaid, New Taipei, Taiwan) according to the manufacturer’s procedure. RNA samples were treated with DNase to remove the DNA contamination using the RapidOut DNA removal kit (Thermo, Waltham, MA, USA). PCR was used to check the RNA quality before real-time RT-PCR. The first-strand cDNA was synthesized from 1 μg of each indicated DNA-free RNA sample by using the random hexamer as a primer and other components of the cDNA synthesis kit (Invitrogen, Waltham, MA, USA) according to the manufacturer’s procedure. To quantitatively measure the relative expression of nuclear or organellar genes, real-time RT-PCR was performed with nuclear *EF-1α* as a control. The PCR reactions involved a 20 μL volume containing 10 μL GoTaq qPCR Master Mix (Promega, Madison, WI, USA), 1 μL cDNA as a template and the indicated primers (Supplementary Table S1) at a final concentration of 100 nM using the CFX connect Real-Time System with the parameters described above. The qRT-PCR assays were conducted with the biological replicates and for each with triplicate reaction. 

### 2.4. RNA-Seq Analysis

The raw sequence reads data of RNA-Seq from the leaf tissues of a PEP-deficient tobacco line (M0) and untransformed wild-type tobacco were retrieved from the Sequence Read Archive with the BioSample accession numbers SRR12417980 and SRR12418005, respectively [25]. The statistics of total reads, along with the average read length and total number of bases between PEP-deficient (M0) tobacco and wild-type plants, are summarized in Supplementary Tables S2 and S3. The comparative analysis of the organellar transcriptome of the PEP-deficient (M0) tobacco and the wild-type plants involved the use of CLC Genomics Workbench 10. In brief, the RNA-Seq reads were mapped to the cpDNA (NC_001879) or mtDNA (NC_006581) templates, respectively, with the indicated mapping parameters (Supplementary Tables S2 and S3). In addition, the reads per kilobase of exon model per million mapped reads (RPKM) values were analyzed with the default parameters and normalized to that of nuclear *EF-1α* to estimate the relative gene expression profile [26,27,28]. 

To analyze the nuclear transcriptome, the reads were mapped onto the nuclear genome of *Nicotiana tabacum* using the TopHat2 algorithm [29] with the default parameters. The reference genome can be found on the NCBI website (ftp://ftp.ncbi.nlm.nih.gov/genomes/all/GCF/000/715/135/GCF_000715135.1_Ntab-TN90/GCF_000715135.1_Ntab-TN90_genomic.fna.gz (accessed on 16 January 2020). The gene expression levels were estimated using HTSeq software in union mode [30]. The differential expression of genes (DEGs) between samples were analyzed by DEGseq, with TMM as a normalization method [31]. The DEGs were defined with an absolute value of log2 fold changes (FC) ≥1 and a q-value ≤0.005. The DEGs were clustered according to their functionality using eggNOG-mapper [32]. The enrichment of the metabolic pathways for the DEGs were predicted using the Kyoto Encyclopedia of Genes and Genomes (KEGG) automated annotation server with the default parameters [33]. 

### 2.5. DNA-Seq Analysis

The raw reads of DNA-Seq from the leaf tissues of four transgenic tobacco lines (M0, M8, M17 and M29) with overexpression of TALEN targeting the plastid *rpoB* gene, which were sequenced by the Illumina HiSeq X Ten platform [25] were retrieved from the NCBI Sequence Read Archive (http://trace.ncbi.nlm.nih.gov/Traces/sra/ (accessed on 7 August 2020) with the BioSample accession numbers SRR12407060, SRR12407063, SRR12407106 and SRR12407170, respectively. In addition, the DNA-Seq reads (SRR955758) of the untransformed tobacco cultivar TN90 were retrieved from the NCBI database [34]. The statistics of the total reads along with the total number of bases were summarized in Supplementary Table S4. The comparative analysis of mtDNA among the plants involved the use of the CLC Genomics Workbench 10 as described [27], with modifications. In brief, the raw DNA-Seq reads were mapped to mtDNA (NC_006581) with the mapping parameters of length fraction (L) = 1.0 and similarity (S) = 1.0, using the CLC Genomics Workbench 10 and normalized to those of nuclear *EF-1α* to estimate the relative mtDNA copy number. 

## 3. Results and Discussion

### 3.1. Chloroplast Gene Expression in PEP-Deficient Plants

Previously, deletion of the *rpoA, rpoB*, *rpoC1* or *rpoC2* genes, encoding the subunits of PEP in tobacco, by plastid transformation [13,14,15] or loss of the functional *rpoB* operon by using cpTALEN-mediated technology [25] resulted in the loss of autophototrophic growth in plants but still retained the capacity for heterotrophic growth. In the absence of PEP activity, mRNAs initiating from the PEP promoters would be absent and the transcripts would be initiated exclusively from the NEP promoters. Previous studies based on run-on assays, RNA gel blotting and nylon filter macroarray have shown that all parts of the cpDNA are transcribed in tobacco with the disruption of PEP genes, though distinct expression profiles were observed between wild-type and PEP-deficient plastids [6,18], suggesting that NEP plays an important role in plastid transcription. However, hybridization-based technologies have limitations for high-throughput studies (e.g., the availability of the gene numbers being investigated) and their resolution is not at the nucleotide level. 

**Figure 1:**
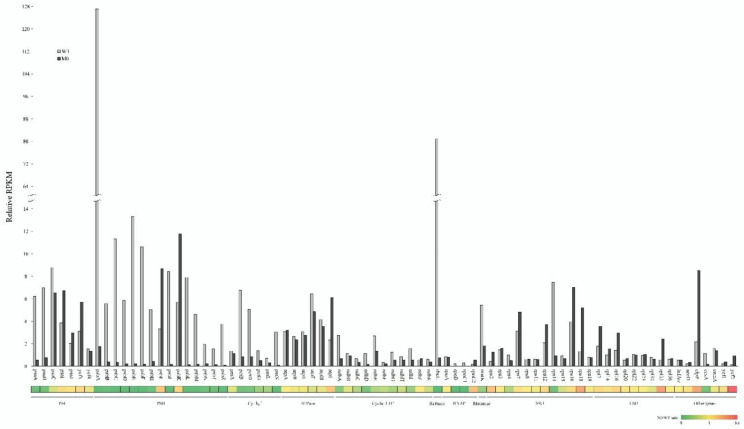
The relative expression levels of plastid protein-coding genes between PEP-deficient tobacco and wild-type plants. The relative RPKM analysis was used to measure the relative expression levels of plastid protein-coding genes by mapping the RNA-Seq reads from the leaf tissues of the PEP-deficient (M0) tobacco and wild-type (WT) plants to the cpDNA (NC_001879) template using the CLC Genomic Workbench with the parameters of length fraction (L) = 0.8 and similarity (S) = 0.8, with an exception for petN (L = 0.7; S = 0.8). The RPKM of the nuclear EF-1α transcripts was used as a control for normalization, set to 1. The ratio of relative RPKM between M0 and the WT is illustrated as a heatmap at the bottom. PSI and PSII, Photosystem I and II; Cyt b6/f, Cytochrome b6/f complex; ATPase, ATP synthase complex; cyclic ETC, cyclic electron transport chain; Rubisco, ribulose 1,5-bisphosphate carboxylase/oxygenase; RNAP, RNA polymerase; SSU, ribosomal small subunits; LSU, ribosomal large subunits.

In this study, we took advantage of RNA-Seq of the leaf tissue to investigate the steady state of genome-wide changes in the plastid gene expression of the transgenic tobacco (M0) line with the disruption of PEP mediated by TALEN, and compared it with the wild-type (Figure 1). A significantly altered pattern of plastid gene expression was observed between PEP-deficient (M0) tobacco and wild-type plants when the RNA-Seq reads were mapped to the cpDNA template (Supplementary Figure S1). This suggests that the dramatic change in the plastid gene expression profile might occur in PEP-deficient (M0) plants. Previously, almost no RNA-Seq reads covered the *rpoB* and *rpoC1* genes in M0 plants, with no significant change for *rpoA* being reported [25]. Additionally, the bioinformatic analysis as well as the RT-PCR and qRT-PCR assays showed the very dramatic reduction in the *rpoB* and *rpoC1* transcripts but no significant change in *rpoA* mRNA in PEP-deficient (M0) plants compared with that of the wild-type (Figure 1) [25]. Previously, the transcription of the *rpoB* operon (*rpoB*-*C1*-*C2*) in tobacco was exclusively conferred by NEP [4,17]. In this study, on the basis of the comparative RNA-Seq analysis of leaf tissues from PEP-deficient (M0) tobacco and wild-type plants, we strongly suggest that the transcription of the *rpoA* gene is also mainly carried out by NEP in tobacco (Figure 1). In addition, the microhomology-mediated recombination of *rpoC2* and *ycf2* to create the *ycf2*-*rpoC2* chimeric gene [25] is attributed to the increase in *rpoC2* transcripts (Figure 1)*,* particularly those in the form of *ycf2*-*rpoC2* chimeric transcripts in PEP-deficient (M0) plants compared with wild-type tobacco. The transcription of *ycf2* in dicot plants is mainly achieved by the NEP [4]; therefore, the significant level of *ycf2*-*rpoC2* chimeric transcripts [25] is initiated only by the NEP promoter in PEP-deficient (M0) plants.

We took advantage of the RNA-Seq approach to further study the steady-state expression of other plastid protein-coding genes and the roles of PEP and NEP in their transcription. The genome-wide expression levels of plastid protein-coding genes was revealed through the relative RPKM by normalization to that of nuclear *EF-1α* mRNA for a comparison between PEP-deficient (M0) tobacco and wild-type plants (Figure 1). In addition, the ratio of relative RPKM was shown as a heat map. Most plastid genes were transcribed, with the exception of the *rpoB* and *rpoC1* genes, as mentioned above, in PEP-deficient (M0) plants. The results suggested that NEP plays an essential role in plastid biogenesis, which is consistent with a previous study using a macroarray [18]. The relative RPKM levels of transcripts encoding for the subunits of PSI (*psaA* and *psaB*), PSII (except *psbJ* and *psbK*), cytochrome b_6_/f complex (except *petA*), cyclic ETC complex (except *ndhB* and *ndhJ*), and *rbcL, ccsA*, *matk*, *rps4* and *rps14* transcripts were significantly higher (>1.5-fold) in the wild-type than in PEP-deficient (M0) tobacco. In particular, the levels of *psbA* and *rbcL* were more than 72- and 104-fold higher, respectively, in the wild-type than in PEP-deficient (M0) plants (Figure 1). This analysis suggested that the above-mentioned genes are mainly transcribed by PEP. In barley, most plastid genes, including genes coding for photosynthesis-related proteins, have both PEP and NEP promoters [19]. The *psaA*, *psaB* and *rps14* genes are organized as an operon and are mainly transcribed by the PEP promoter [6,16]. Further analysis revealed that photosynthesis-related transcripts such as *psaA* and *psaB* were detectable, though in reduced amounts in PEP-deficient tobacco (the transplastomic *rpoA* mutant); however, the size of their transcripts was altered because of the switching of promoters from PEP to the NEP promoter further upstream [18]. In this study, the levels of *psaA*, *psaB* and *rps14* transcripts were 8- to 11-fold higher in wild-type plant than in PEP-deficient (M0) tobacco, which was approximately consistent with their transcription as an operon, and were initiated mainly by the PEP promoter and to a lesser extent, by the NEP promoter. However, immunoblot analysis showed no detectable signals of photosynthesis-related proteins in the PEP-deficient plastids [18], suggesting that post-transcriptional regulation plays an important role in protein accumulation. In this study, as indicated by RNA-Seq analysis, the steady state of *rbcL* mRNA in PEP-deficient (M0) plants was significantly reduced (104-fold lower), which is consistent with a previous study showing that the *rbcL* gene is transcribed by a PEP promoter in tobacco, since the accumulation of *rbcL* transcripts in each deletion mutant of the *rpoA, B, C1* or *C2* was significantly reduced (25-fold or 2–3 fold lower) according to the gel blots [14] or macroarray [18]. However, the accumulation of *rbcL* transcript residues derived from the processed read-through transcripts was observed in PEP-deficient tobacco [14,18], which were transcribed by NEP. In white leaves of the barley albostrians mutant, in which the plastid is ribosome-deficient and lacks all the plastid-encoded proteins including PEP, the *rbcL* gene is transcribed by a NEP promoter [19].

In contrast, the relative RPKM level of *psaI*, *ycf3*, *psbI*, *psbK, atpI*, *rps2*, *rps7*, *rps12*, *rps16*, *rps18*, *rpl2, rpl14, rpl16, rpl33, accD, clpP*, *ycf1* and *ycf2* transcripts were significantly higher (>1.5-fold) in PEP-deficient (M0) plants than in the wild-type. In particular, that of *ycf2, rpl33, clpP* and *rps18* were increased by up to 6.6-, 4.2-, 4.0- and 3.9-fold, respectively, in M0 plants as compared with the wild-type (Figure 1). Previously, a qRT-PCR assay also confirmed the higher level of *ycf2* transcripts in PEP-deficient (M0) plants than in the wild-type [25]. The findings suggested that the above-mentioned genes are mainly transcribed by the NEP, and the enhanced level of NEP, particularly *rpoT3*, in PEP-deficient tobacco (see below) might significantly increase the transcription of these genes mainly through the action of NEP promoters. Previously, *accD* and *clpP* revealed higher steady-state levels of the transcripts for each *rpo* deletion in mutant plants than in the wild-type [8,13,14,16], which is consistent with our study. In addition, overexpression of the *RpoT3* gene from *Arabidopsis* or *N. sylvestris* in tobacco revealed the enhanced transcription of certain genes by NEP promoters [8].

Transcription of the other protein-coding genes would expect to be mainly carried out by NEP, since the transcript levels show no significant change (<1.5-fold) between PEP-deficient (M0) plants and wild-type tobacco. Alternatively, the increase in NEP activity (see below) to compensate for the loss of PEP activity in PEP-deficient (M0) tobacco would possibly mask the important role of PEP in the regulation of gene expression in this study. Switching of the promoter from PEP to NEP was demonstrated in *Arabidopsis* with mutations of the sigma factor [35,36].

In summary, our results suggested that along with most housekeeping genes, NEP also plays an important role in regulating the expression of genes involved in photosynthesis such as PSI (*psaC*, *psaI*, *psaJ*, *ycf3* and *ycf4*), PSII (*psbI* and *psbK*), the cytochrome b_6_/f complex (*petA*), the ATP synthase complex (*atpA*, *atpB*, *atpE*, *atpF, atpH* and *atpI*) and the cyclic ETC complex (*ndhB* and *ndhJ*), and in the transcription of *rpoA*. In contrast, in addition to the photosynthesis-related genes, PEP also played an important role in regulating the expression of genes involved in housekeeping functions (e.g., *matK*, *rps4*, *rps14* and *ccsA*). Transplastomic plants offer several advantages in metabolic engineering and synthetic biology [37]. This study can provide useful information regarding the use of promoters to drive transgene expression, particularly in non-photosynthetic tissues for future plastid transformation as well as synthetic biology.

### 3.2. Increase in the Mitochondrial DNA Copy Number and Gene Expression in PEP-Deficient Plants

**Figure 2:**
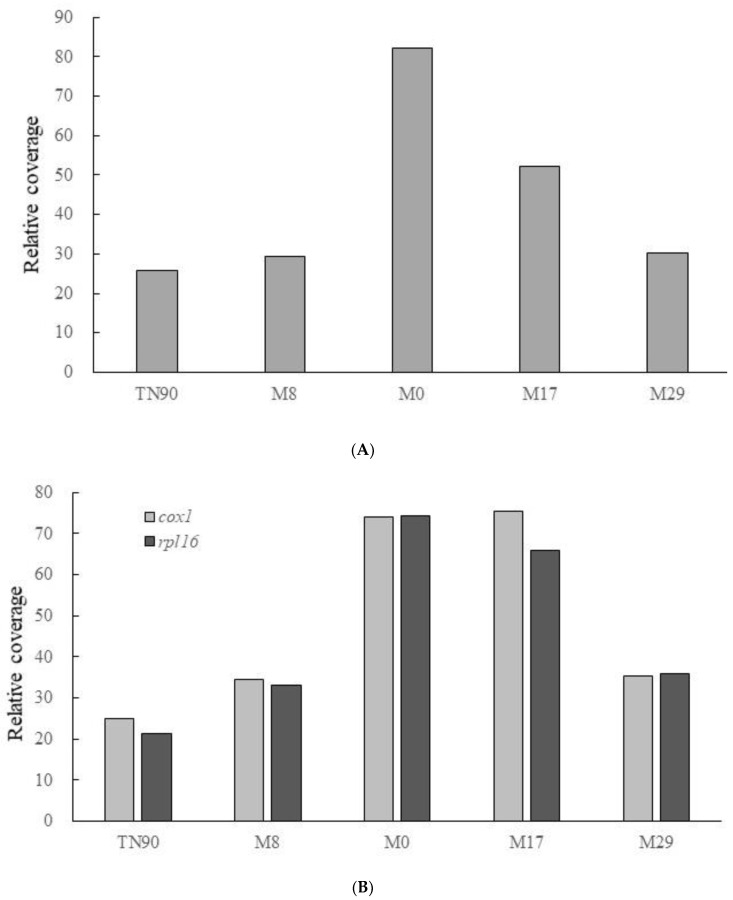

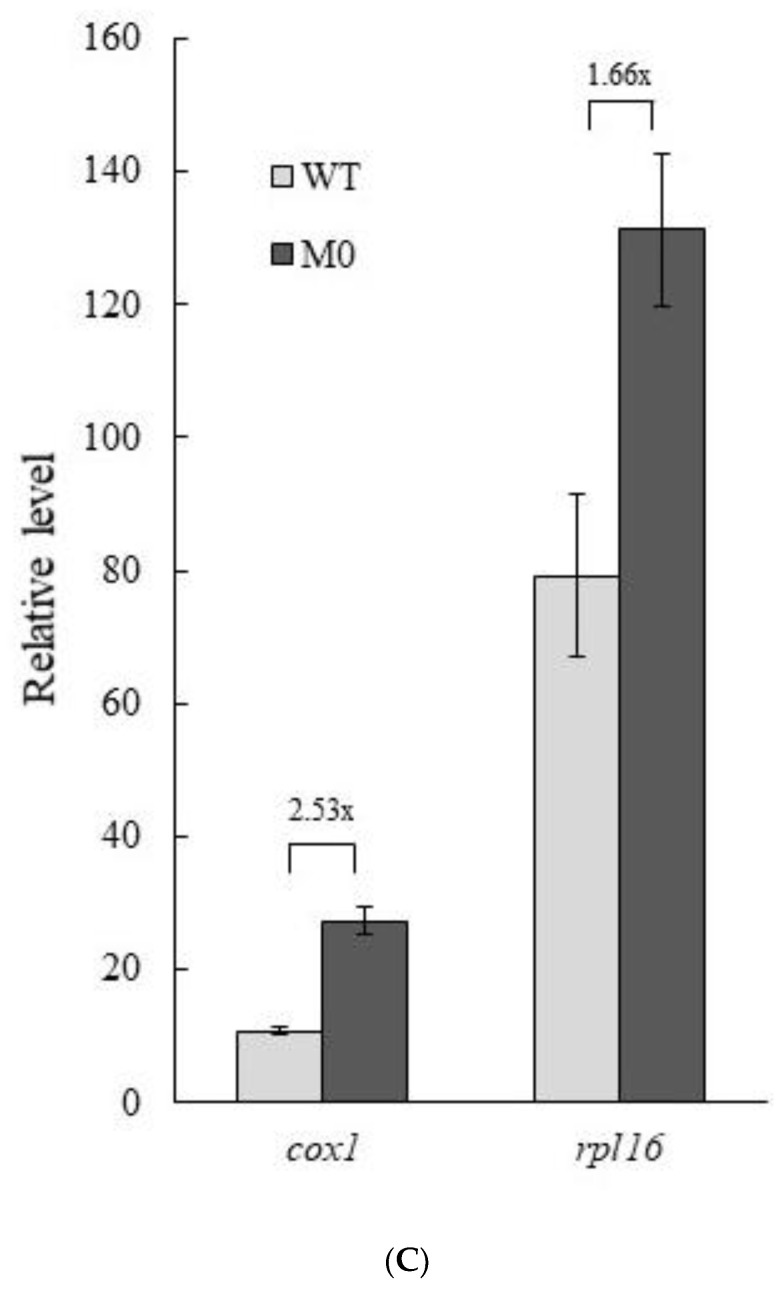
The relative copy numbers of mitochondrial DNA in transgenic tobacco lines. (**A**). The DNA-Seq reads from transgenic M0, M8, M17, M29 and untransformed TN90 plants were mapped to the mtDNA template (NC_006581) by the CLC Genomic Workbench 10 with the most stringent parameters of L = 1.0 and S = 1.0 (Supplementary Table S4). The relative average coverage of bulk mtDNA in transgenic (M8, M0, M17 and M29) and untransformed TN90 plants is shown by the normalization of the average coverage of each mtDNA to that of the nuclear *EF-1α* gene in the corresponding plants. (**B**). The relative average coverage of the mitochondrial *cox1* and *rpl16* genes in transgenic (M8, M0, M17 and M29) and untransformed TN90 plants is shown by the normalization of the average coverage of each gene to that of the nuclear *EF-1α* gene in the corresponding plants. (**C**). The relative copy number of mitochondrial genes was estimated by a qPCR assay in PEP-deficient (M0) tobacco and wild-type (WT) plants. Total DNA was isolated from the leaf tissues of M0 plants (dark grey) and WT tobacco (light grey) grown in half-strength MS media. The relative copy numbers of the mitochondrial *cox1* and *rpl16* genes were estimated by qPCR with nuclear *EF-1α* as a control. The qPCR assay was carried out in triplicate. Data are means ± SD.

Chloroplasts and mitochondria have close and complex metabolic interdependencies [38]. Therefore, the disruption of the plastid function might directly or indirectly affect mitochondrial biogenesis. In albostrians mutants of barley, which lack the plastid translational apparatus (ribosome), both the mitochondrial transcript levels and gene copy numbers were enhanced in photosynthetically incompetent white leaves compared with green leaves [39]. Previously, overexpression of the recombinant TALEN targeting the plastid *rpoB* gene caused distinctive phenotypes within transgenic tobacco lines, ranging from no apparent phenotypic effect (M8 line) to weakly (M29) or severely (M17) variegated chlorosis to complete albinos (M0) [25]. The analysis of the DNA-Seq reads from four transgenic lines (M0, M8, M17 and M29 plants) with distinct phenotypes revealed the different levels of the altered cpDNA structure [25]. To investigate the effect of mtDNA in transgenic plants, in this study, the DNA-Seq reads were mapped to tobacco mtDNA with the highest stringency (Supplementary Table S4; Supplementary Figure S2). The copy number of bulk mtDNA in the transgenic plants was estimated according to the relative average coverage of mtDNA in each plant, with normalization using the nuclear *EF-1α* gene (Figure 2A). The copy number of mtDNA in M0 and M17 plants increased by about 2.8- and 1.8-fold, respectively, compared with the M8 and M29 plants (Figure 2A). For the mitochondrial *cox1* and *rpl16* genes, the copy number was bioinformatically estimated at about 2- to 2.2-fold higher in M0 and M17 plants than in M8 and M29 plants (Figure 2B). To confirm the results of the bioinformatic analysis, a qPCR assay was further carried out to measure the relative amount of *cox1* and *rpl16* gene between PEP-deficient (M0) tobacco and the untransformed wild-type with nuclear *EF-1α* as a control. The copy number of mitochondrial *cox1* and *rpl16* genes was higher by about 2.5- and 1.7-fold, respectively, in PEP-deficient (M0) plants than in the wild-type (Figure 2C), which is approximately consistent with the bioinformatic analysis. Our results demonstrated that the mtDNA copy number was up-regulated in tobacco plants with disruption of the function of plastid PEP.

**Figure 3:**
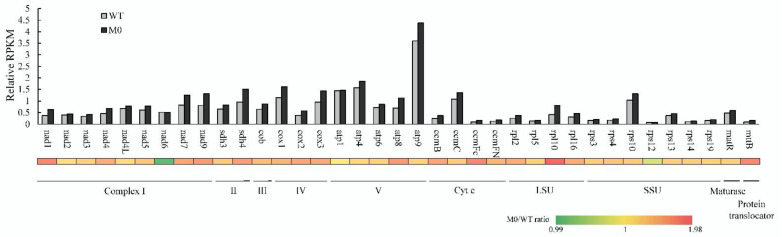
The relative expression levels of mitochondrial protein-coding genes in PEP-deficient tobacco and wild-type plants. The relative RPKM analysis was used to measure the relative level of mitochondrial protein-coding transcripts by mapping the RNA-Seq reads from the leaf tissues of the PEP-deficient (M0) tobacco and wild-type (WT) plants to the mtDNA (NC_006581) template using the CLC Genomic Workbench 10 with the parameters of L=0.8 and S=0.8. The RPKM of the nuclear *EF-1α* transcripts was used as a control for normalization, set to 1. The ratio of relative RPKM between M0 and the WT is illustrated as a heatmap at the bottom. Complex I, II, III, IV and V, respiratory electron transport chain complex I, II, III, IV and V (ATP synthase complex); Cyt c, cytochrome C biogenesis; LSU, ribosomal large subunits; SSU, ribosomal small subunits.

To investigate the relative expression levels of mitochondrial genes between PEP-deficient (M0) tobacco and untransformed wild-type plants, in this study, the RNA-Seq reads were mapped to tobacco mtDNA with high stringency (Supplementary Table S3; Supplementary Figure S3), and the relative RPKM of mitochondrial protein-coding genes was analyzed with normalization to that of the nuclear *EF-1α* gene. In the results of the relative RPKM analysis of mitochondrial protein-coding genes, the expression of most genes was slightly increased (<2-fold) in PEP-deficient (M0) plants compared to the wild-type, with the exception of *atp1*, *nad6* and *rps12*, which were almost unchanged (Figure 3, Supplementary Table S3). The results of the RPKM analysis were approximately consistent with a previous study on mitochondrial gene expression based on a qRT-PCR assay, in which the relative expression levels of both *cox1* and *rpl16* genes was approximately 1.7-fold higher in PEP-deficient (M0) plants than in the wild-type [25]. The slightly higher expression level of mitochondrial transcripts might be caused by the slightly increased amount of their corresponding DNA, as discussed above (Figure 2; Supplementary Table S4). Alternatively, since mtDNA do not encode any known protein subunits involved in transcription, and no activity of eubacteria-like RNA polymerase was reported in the mitochondria of land plants [3,36], the slightly increased expression of nucleus-encoded bacteriophage T7-like RNA polymerase genes (*rpoT1* and *rpoT2*) with the encoded proteins targeting the mitochondria might contribute to the slightly higher level of mitochondrial transcripts in PEP-deficient (M0) plants (see below). Nevertheless, our results suggested that the mitochondrial gene copy number and gene expression levels were slightly affected by retrograde signaling from the plastid of the PEP-deficient (M0) tobacco.

### 3.3. The Accumulation of Nuclear RpoT mRNA Was Altered in PEP-Deficient Plants

**Figure 4:**
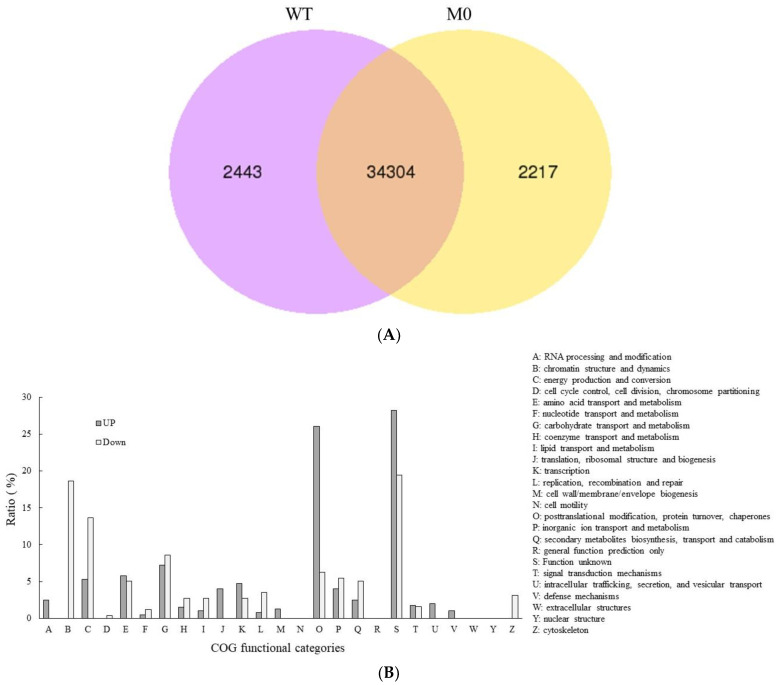
The functional classification based on clusters of orthologous groups (COG) for differentially expressed genes between PEP-deficient tobacco and wild-type plants. (**A**). A Venn diagram of the differentially expressed genes is shown. The sum of the numbers in each circle is the total number of genes expressed in PEP-deficient (M0) tobacco or wild-type (WT) plants. The overlapping region represents the genes expressed in common in both M0 and WT tobacco. (**B**). The ratio of up-regulated (grey bar) or down-regulated (white bar) DEGs in each annotated COG functional category between PEP-deficient (M0) tobacco and wild-type (WT) plants. The COG functional categories are shown on the right.

The PEP-deficient plants generated by either the transplastome- or TALEN-mediated approach exhibited the albino phenotype [4,13,15,25], which suggests a dramatic change in nuclear gene expression. In this study, RNA-Seq reads were used to investigate the relative expression levels of nuclear genes in PEP-deficient (M0) tobacco and untransformed wild-type plants. There were 34,304 commonly expressed genes, with an additional 2217 and 2443 uniquely expressed genes for PEP-deficient (M0) tobacco and wild-type plants, respectively (Figure 4A). Of these, 767 genes, 466 up-regulated and 301 down-regulated, were statistically screened as differentially expressed genes (DEGs) between PEP-deficient (M0) tobacco and wild-type plants (Supplementary Figure S4). The functional classification based on clusters of orthologous groups (COG) revealed that genes with an unknown function account for the largest portion of the DEGs (Figure 4B). Additionally, among those with known functional groups, genes involved in posttranslational modification, protein turnover and chaperone function contributed the highest percentage of up-regulated genes in PEP-deficient (M0) tobacco compared with wild-type plants, followed by genes involved in carbohydrate transport and metabolism, and amino acid transport and metabolism (Figure 4B). In contrast, genes involved in chromatin structure and dynamics, energy production and conversion, carbohydrate transport and metabolism ranked as the top three highest functional groups contributing to the down-regulated expression of genes in PEP-deficient (M0) tobacco compared with wild-type plants (Figure 4B). In the analysis of the metabolic pathways using KEGG, the significant down-regulation of key genes involved in chlorophyll biosynthesis and binding, such as protochlorophyllide oxidoreductase, chlorophyllide a oxygenase and chlorophyll a/b binding proteins, might explain the albino phenotype as well as the loss of photosynthetic capability in PEP-deficient (M0) plants (Supplementary Figure S5).

**Figure 5:**
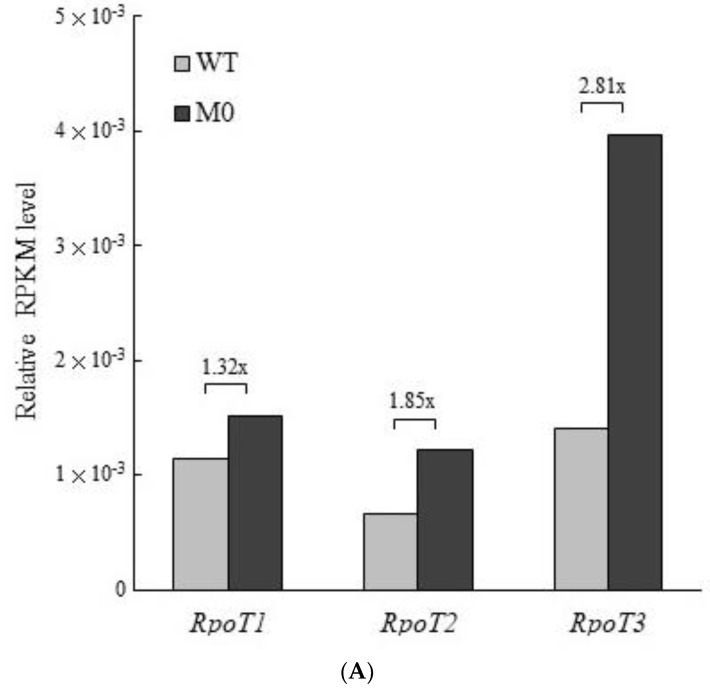

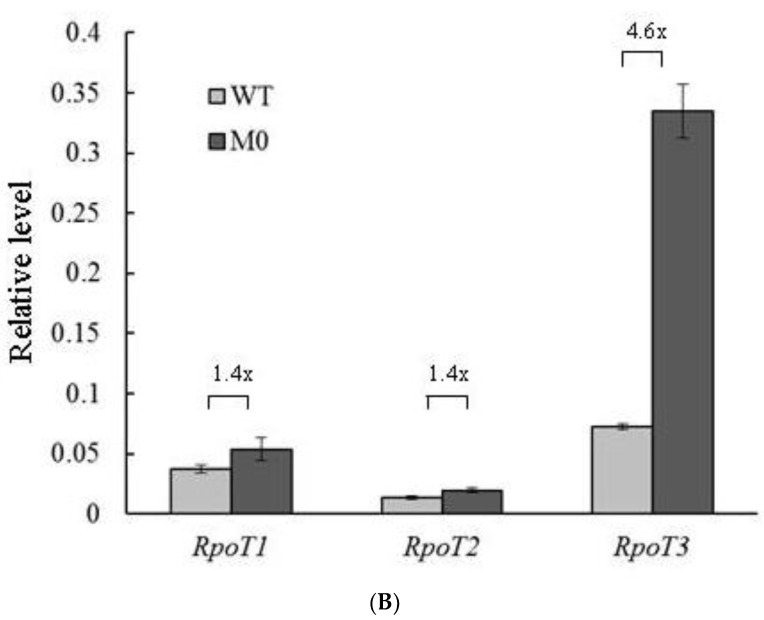
The relative expression levels of nucleus-encoded *RpoT* genes between PEP-deficient tobacco and wild-type plants. (**A**). The relative RPKM analysis was used to measure the relative expression levels of nucleus-encoded *RpoT* (*RpoT1*, *RpoT2* and *RpoT3*) genes by mapping the RNA-Seq reads from the leaf tissues of the PEP-deficient (M0) tobacco and wild-type (WT) plants to the corresponding templates using the CLC Genomic Workbench 10 with the parameters of L = 0.8 and S = 0.8. The RPKM of nuclear *EF-1α* transcripts was used for normalization, set to 1. (**B**). A qRT-PCR assay with the gene-specific primers (Supplementary Table S1) was used to measure the relative levels of *RpoT1*, *RpoT2* and *RpoT3* transcripts between the PEP-deficient (M0) tobacco and wild-type (WT) plants, with nuclear *EF-1α* mRNA as a control. The qRT-PCR assay was carried out in triplicate. Data are means ± SD.

The tobacco nuclear genome encodes three phage-type RNA polymerase genes (*RpoT1*, *RpoT2* and *RpoT3*), with RPOT1 and RPOT3 exclusively targeting the mitochondria and chloroplasts, respectively, while RPOT2 targets both organelles [7]. A previous study based solely on a qRT-PCR assay showed that the expression of *RpoT3* transcripts was increased by 2.6-fold in the leaf tissues of transplastomic tobacco with deletion of the *rpoA* gene compared with that of the wild-type [7], suggesting retrograde signaling from the plastid to the nucleus. In this study, on the basis of the RNA-Seq analysis, we studied whether the steady-state gene expression of *RpoT* was altered in PEP-deficient (M0) plants. According to the relative RPKM analysis, the steady state of *RpoT3* transcripts was highest and was up to 2.1- and 1.2-fold higher than that of *RpoT2* and *RpoT1* mRNA, respectively, in the leaves of wild-type tobacco (Figure 5A). In addition, the expression of the *RpoT1*, *RpoT2* and *RpoT3* genes was higher by about 1.32-, 1.85- and 2.81-fold, respectively, in PEP-deficient (M0) plants than in wild-type tobacco, according to the relative RPKM analysis (Figure 5A). The qRT-PCR assay was carried out to further confirm the results of the RNA-Seq analysis. The results showed the highest expression level of *RpoT3* transcripts in the leaves of wild-type tobacco plants, which was up to 5.6- and 1.9-fold higher than that of *RpoT2* and *RpoT1* mRNA, respectively. Furthermore, the qRT-PCR results were approximately consistent with those of the relative RPKM analysis, with the mRNA levels of *RpoT1* as well as *RpoT2* slightly increased by about 1.4-fold, and that of *rpoT3* transcripts being significantly higher, up to 4.6-fold, in PEP-deficient (M0) plants than in the wild-type (Figure 5B). Our results strongly demonstrated that the retrograde signaling from the plastid of PEP-deficient (M0) tobacco up-regulated the expression of all three nuclear-encoded phage-type RNA polymerase (NEP or *RpoT*) genes, whose function is involved in the transcription of chloroplasts as well as mitochondria. In *Arabidopsis*, *RpoT2* (*RpoTmp*) and *RpoT3* (*RpoTp*) have overlapping as well as gene-specific functions in the transcription of plastid genes [10]. The *RpoT3* transcripts were much more abundant than those of *RpoT2* in the leaf tissues of tobacco (Figure 5) [7]; however, their corresponding functional roles in PEP-deficient (M0) tobacco could not be distinguished in this study. A previous report revealed that overexpression of the *RpoT3* (*RpoTp*) gene from *Arabidopsis* or *N. sylvestris* driven by the constitutive CaMV 35S promoter in tobacco could enhance the transcription of *atpB* through increasing the use of NEP promoters and decreasing the use of PEP promoters [8], though only a limited number of genes were analyzed. Plastid genes often possess multiple promoters for the regulation of gene expression in response to developmental and environmental cues. In this study, the significant increase in *rpoT3* transcripts for plastid NEP might at least partially compensate for the functional loss of PEP in M0 tobacco. Previously, the promoter switch was demonstrated in a sigma factor (Sig6) knockout mutant of *Arabidopsis*, in which a further upstream NEP promoter could compensate for failed transcription of the *atpB* gene from the main PEP promoter [35,36].

## 4. Conclusions

We used the RNA-Seq approach to study the steady-state level of genome-wide gene expression in PEP-deficient tobacco compared with that of wild-type plants. The steady-state level of transcripts reflects the combinatorial effect of the rate of transcription attributing to the strength and types of promoter usage as well as the relative abundance of RNA polymerases (e.g., NEP and PEP) and their corresponding regulatory factors, and RNA processing kinetics and stability. In a comparative RNA-Seq analysis, all the plastid protein-coding genes were transcribed by NEP, though not equally effective. In addition, most of the house-keeping genes and photosynthesis-related genes were mainly transcribed by NEP and PEP, respectively. However, transcription of the genes involved in PSI (with the exception of *psaA* and *psaB*), PSII (*psbJ* and *psbK*), and the cytochrome b_6_/f complex (*petA*), the ATP synthase complex (all genes) and cyclic electron transport (*ndhB* and *ndhJ*) was also significantly achieved by NEP. In contrast, PEP also played important roles in the transcription of some house-keeping genes (e.g., *matK*, *rps4*, *rps14* and *ccsA*). This study will provide useful information regarding promoter selection to drive transgene expression in non-photosynthetic tissues for plastid transformation as well as synthetic biology in the future. In addition, the mtDNA copy number as well as the gene expression levels were up-regulated in PEP-deficient (M0) plants, which demonstrated the close communication of plastids and mitochondria. The up-regulation of genome-wide mitochondrial gene expression in PEP-deficient (M0) plants might be caused by both the increase in the mtDNA copy number and the expression levels of the nuclear-encoded *rpoT1* and *rpoT2* genes.

## Supplementary Materials

The following are available online at https://www.mdpi.com/article/10.3390/plants11212860/s1, Table S1. The primers used in this study, Table S2. The statistics of RNA-Seq reads mapped to reference cpDNA template, Table S3. The statistics of RNA-Seq libraries mapped to reference mtDNA template, Table S4. The statistics of DNA-Seq libraries mapped to reference mtDNA template, Figure S1. The distribution pattern and coverage of RNA-Seq reads by mapping to the tobacco cpDNA template between PEP-deficient tobacco and wild-type plants, Figure S2. The distribution pattern and nucleotide coverage of DNA-Seq reads mapped to the tobacco mtDNA among transgenic plants, Figure S3. The distribution pattern and coverage of RNA-Seq reads by mapping to the tobacco mtDNA template between PEP-deficient tobacco and wild-type plants, Figure S4. The differentially expressed genes (DEGs) between PEP-deficient tobacco and wild-type plants, Figure S5. The KEGG pathway enrichment analysis for the differentially expressed genes (DEGs) between PEP-deficient tobacco and wild-type plants.
