# Supplementary material for: A Transcriptomic Analysis of Tobacco Leaf with the Functional Loss of the Plastid rpoB Operon Caused by TALEN-Mediated Double-Strand Breakage
Source: Plants (Basel). 2022 Oct 26;11(21):2860. doi: 10.3390/plants11212860 (PMC9659210; doi:10.3390/plants11212860)
Supplement: Supplementary file 1 [file plants-11-02860-s001.zip › plants-1897333-supplementary.pdf]

Supplementary Table S1. The primers used in this study.

Name	Sequence (5' to 3')	Purpose
cox1-F	GGACGGTCTATCCGCCCTTA	qPCR assay for mitochondrial *cox1* gene
cox1-R	GTCACTAGAACGGACCACACAAA	qPCR assay for mitochondrial *cox1* gene
rpl16-F	TTCTGCGGAAGTATCTACTCGTT	qPCR assay for mitochondrial *rpl16* gene
rpl16-R	GCGCTGCTTCAATGGCTC	qPCR assay for mitochondrial *rpl16* gene
RpoT1-F	TGTGATGAAGGGAAGAACCGC	qRT-PCR assay for nuclear *rpoT1* gene
RpoT1-R	GTGTTTCTTCCAAGTCACCACTCA	qRT-PCR assay for nuclear *rpoT1* gene
RpoT2-F	AAAGCAGCTTATGCTAAGTACTTGT	qRT-PCR assay for nuclear *rpoT2* gene
RpoT2-R	CTTCCTTTTGCTTGTCTTTCTCG	qRT-PCR assay for nuclear *rpoT2* gene
RpoT3-F	GCAGAACGCTGAGACGACCGT	qRT-PCR assay for nuclear *rpoT3* gene
RpoT3-R	TGACTTTGAGCTCCAGTCATATGTT	qRT-PCR assay for nuclear *rpoT3* gene
EF-1α-F	GGTAGGATACAACCCTGATAAAATC	qPCR and qRT-PCR assays for nuclear *EF-1α* gene as normalization
EF-1α-R	CAGTGGGACCAAAAGTCACAAC	qPCR and qRT-PCR assays for nuclear *EF-1α* gene as normalization

Supplementary Table S2. The statistics of RNA-Seq reads mapped to reference cpDNA template.

Library	SRA number	NGS methods	Total reads	Total number of bases	Mapped reads[a]					
					Counts	Percentage of reads	Average length	Number of bases	Percentage of bases	Coverage
WT	SRR12417980	Illumina	48,082,362	7,212,354,300	21,573,633	44.87%	150	3,236,044,950	44.87%	20,743
M0	SRR12418005	Illumina	49,399,502	7,409,925,300	10,524,931	21.31%	150	1,578,739,650	21.31%	10,119

[a]The RNA-Seq reads were mapped to tobacco cpDNA (accession no. NC_001879) using CLC Genomics Workbench 10 with the parameters of length fraction (L) 1.0 and similarity (S) 0.98.

Supplementary Table S3. The statistics of RNA-Seq libraries mapped to reference mtDNA template.

Library	SRA number	NGS methods	Total reads	Total number of bases	Mapped reads[a]					
					Counts	Percentage of reads	Average length	Number of bases	Percentage of bases	Coverage
WT	SRR12417980	Illumina	48,082,362	7,212,354,300	1,605,923	3.34%	150	240,888,450	3.34%	559.09
M0	SRR12418005	Illumina	49,399,502	7,409,925,300	2,719,418	5.50%	150	407,912,700	5.50%	946.85

[a]The RNA-Seq reads were mapped to tobacco mtDNA (accession no. NC_006581) using CLC Genomics Workbench 10 with the parameters of length fraction (L) 1.0 and similarity (S) 0.98.

Supplementary Table S4. The statistics of DNA-Seq libraries mapped to reference mtDNA template.

Library	SRA number	NGS methods	Total reads	Total number of bases	Mapped reads[a]					
					Counts	Percentage of reads	Average length	Number of bases	Percentage of bases	Coverage
TN90[b]	SRR955758	Illumina	310,625,638	31,373,189,438	656,593	0.21%	101	66,315,893	0.21%	154.01
M8	SRR12407060	Illumina	271,083,958	40,933,677,658	469,811	0.17%	151	70,941,461	0.17%	164.75
M0	SRR12407063	Illumina	224,754,856	33,937,983,256	1,885,143	0.84%	151	284,656,593	0.84%	661.07
M17	SRR12407106	Illumina	237,951,144	35,930,622,744	680,191	0.29%	151	102,708,841	0.29%	238.53
M29	SRR12407170	Illumina	243,115,774	36,710,481,874	519,664	0.21%	151	78,469,264	0.21%	182.23

[a]The DNA-Seq reads were mapped to tobacco mtDNA (accession no. NC_006581) using CLC Genomics Workbench 10 with the parameters of length fraction (L) 1.0 and similarity (S) 1.0.

[b]TN90, the DNA-Seq reads of untransformed tobacco cultivar TN90 (Sierro et al., 2014) retrieved from NCBI database was used as control.

Supplementary Figure S1

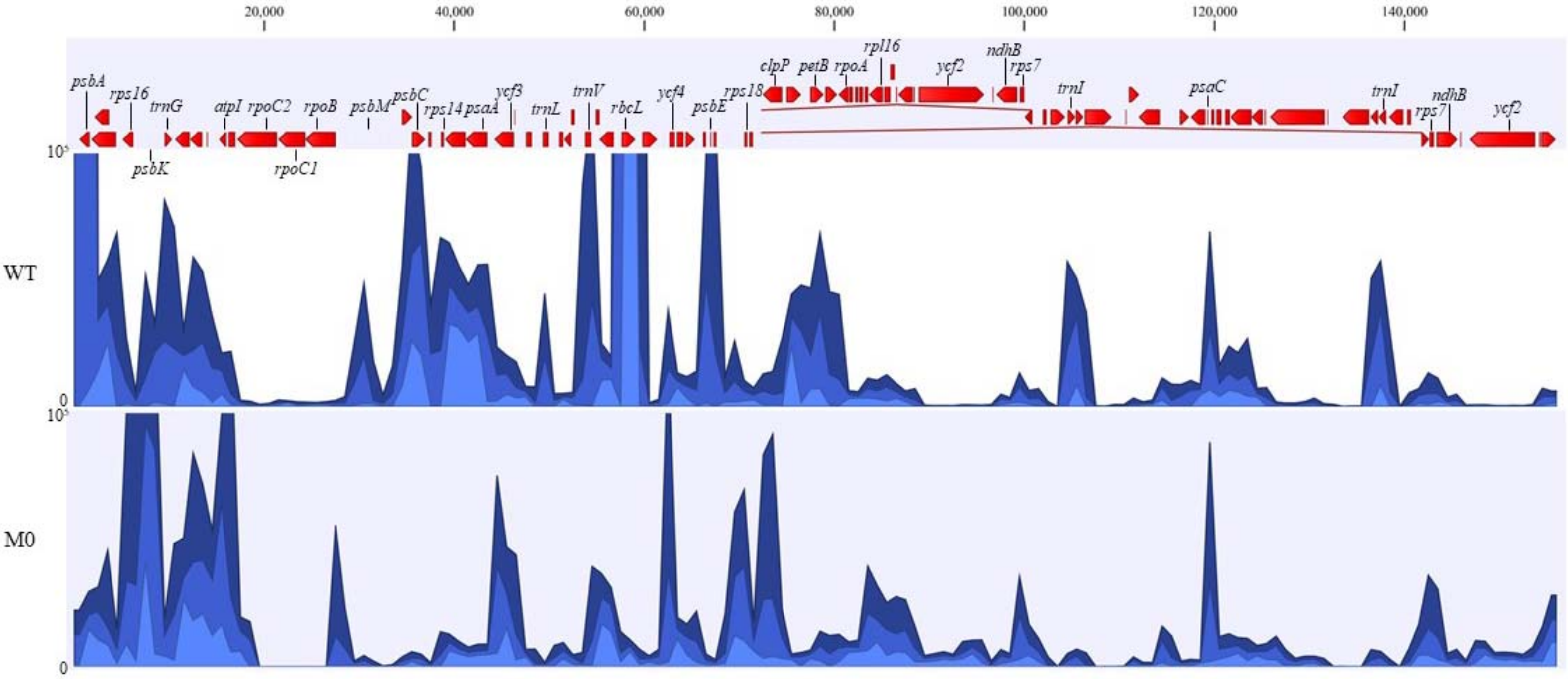

Supplementary Figure S1. The distribution pattern and coverage of RNA-Seq reads by mapping to the tobacco cpDNA template between PEP-deficient tobacco and wild-type plants.

(A) The RNA-Seq reads from PEP-deficient (M0) tobacco and wild-type (WT) plants were mapped to the cpDNA template (NC_001879) by using CLC Genomic Workbench 10 with the high stringent parameters of length fraction 1.0 and similarity 0.98. The detailed mapping statistics of RNA-Seq reads to cpDNA were shown in Supplementary Table 2. The X-axis (top) represents the tobacco cpDNA, and the location of genes were indicated in red color. The Y-axis represents the depth of coverage (altitude of blue color) with the fixed maximum of coverage at 1×10^5. The arrowhead indicates the direction of transcription.

Supplementary Figure S2

A.

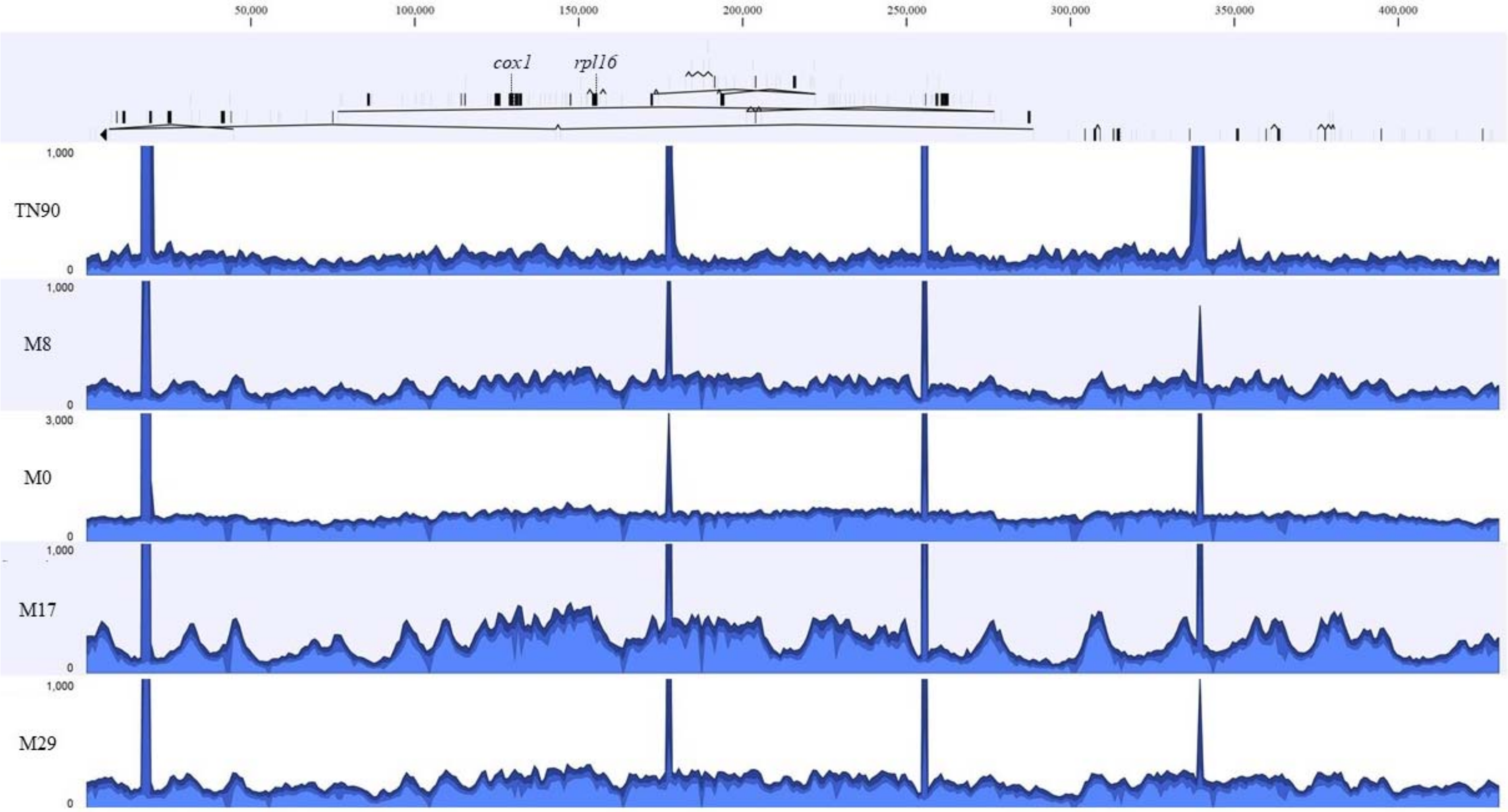

Supplementary Figure S2. The distribution pattern and nucleotide coverage of DNA-Seq reads mapped to the tobacco mtDNA among transgenic plants.

DNA-Seq reads from transgenic M8, M0, M17, M29 and untransformed TN90 plants were mapped to the mtDNA template (NC_006581) by using CLC Genomic Workbench 10 with the most stringent parameters of length fraction (L) 1.0 and similarity (S) 1.0. The detailed mapping statistics of DNA-Seq reads to mtDNA are listed in Supplementary Table 4. The distribution pattern of DNA-Seq reads (blue color) mapped to mtDNA are shown. The number (in bp) on the top of the X-axis is the ruler of mtDNA. The distribution of genes (black) and the direction of transcription (arrow head) in mtDNA with the cox1 and *rpl16* genes indicated. Note, The Y-axis (depth of nucleotide coverage) was not in the same scale between M0 (3,000) and the other plants (1,000).

Supplementary Figure S3

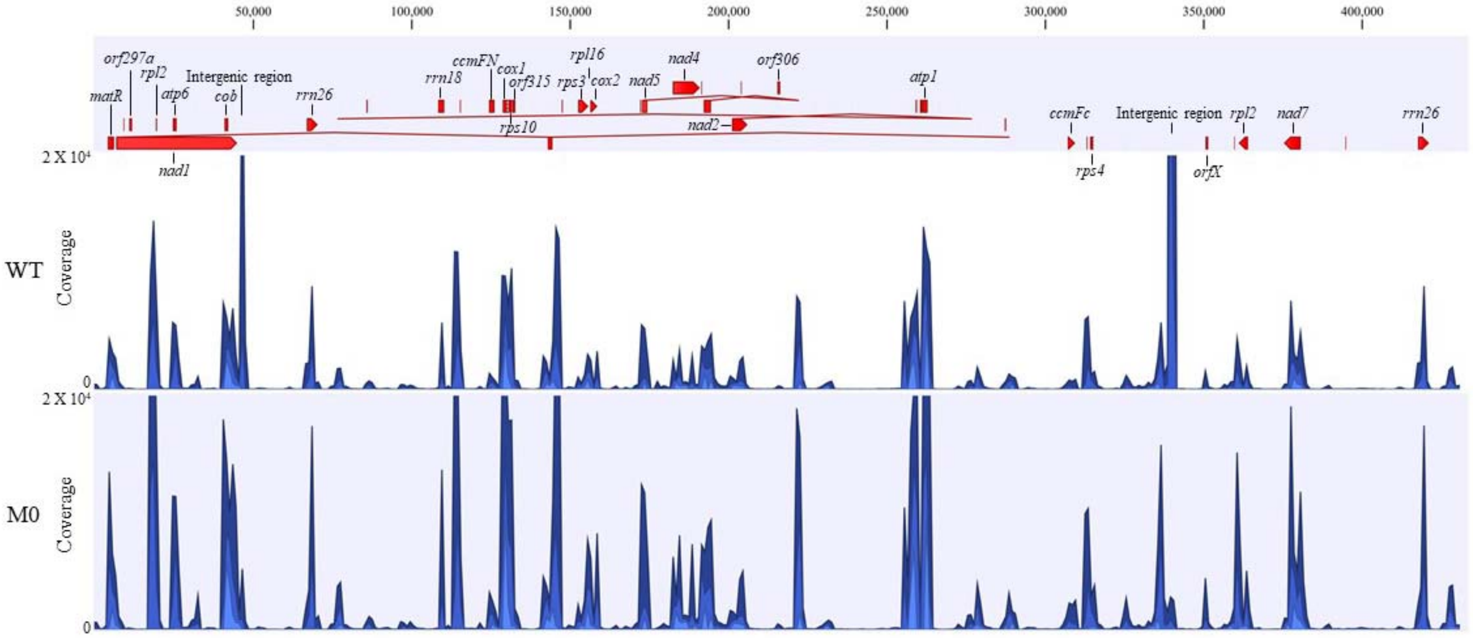

Supplementary Figure S3. The distribution pattern and coverage of RNA-Seq reads by mapping to the tobacco mtDNA template between PEP-deficient tobacco and wild-type plants.

(A) The RNA-Seq reads from PEP-deficient (M0) tobacco and wild-type (WT) plants were mapped to the mtDNA (NC_006581) template by using CLC Genomic Workbench 10 with the high stringent parameters of length fraction 1.0 and similarity 0.98. The detailed mapping statistics of RNA-Seq reads to mtDNA were shown in Supplementary Table 3. The X-axis (top) represents the tobacco mtDNA, and the location of genes were indicated in red color. The Y-axis represents the depth of coverage (altitude of blue color) with the fixed maximum of coverage at $2x10^4$. The arrowhead indicates the direction of transcription.

Supplementary Figure S4

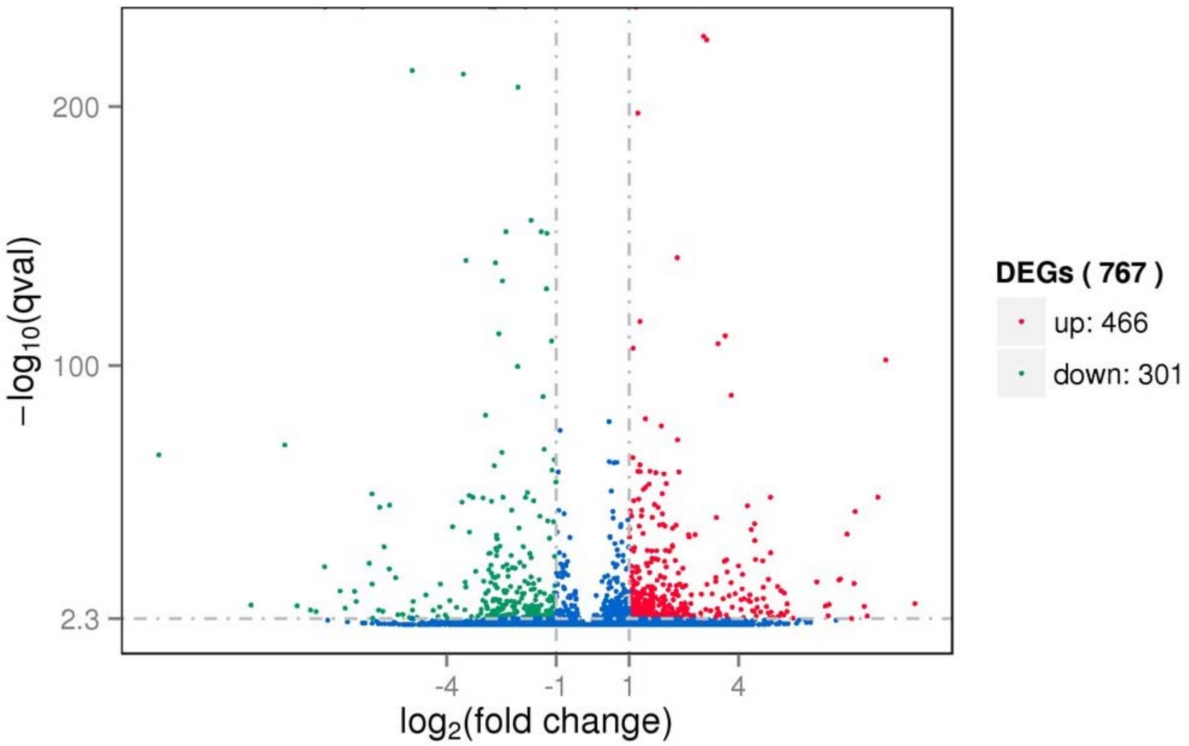

Supplementary Figure S4. The differentially expressed genes (DEGs) between PEP-deficient tobacco and wild-type plants.

The x-axis shows the fold change in gene expression between PEP-deficient (M0) tobacco and wild-type (WT) plants. The y-axis shows the statistically significance of the difference in gene expression. The significantly up- and down-regulated genes are highlighted in red and green, respectively. The genes did not express significantly difference between samples are in blue. The threshold was set as an absolute value of log2 fold changes (FC) ≥1 and a q-value (qval) ≤0.005. The q-value is the p value after normalization.

Supplementary Figure S5.

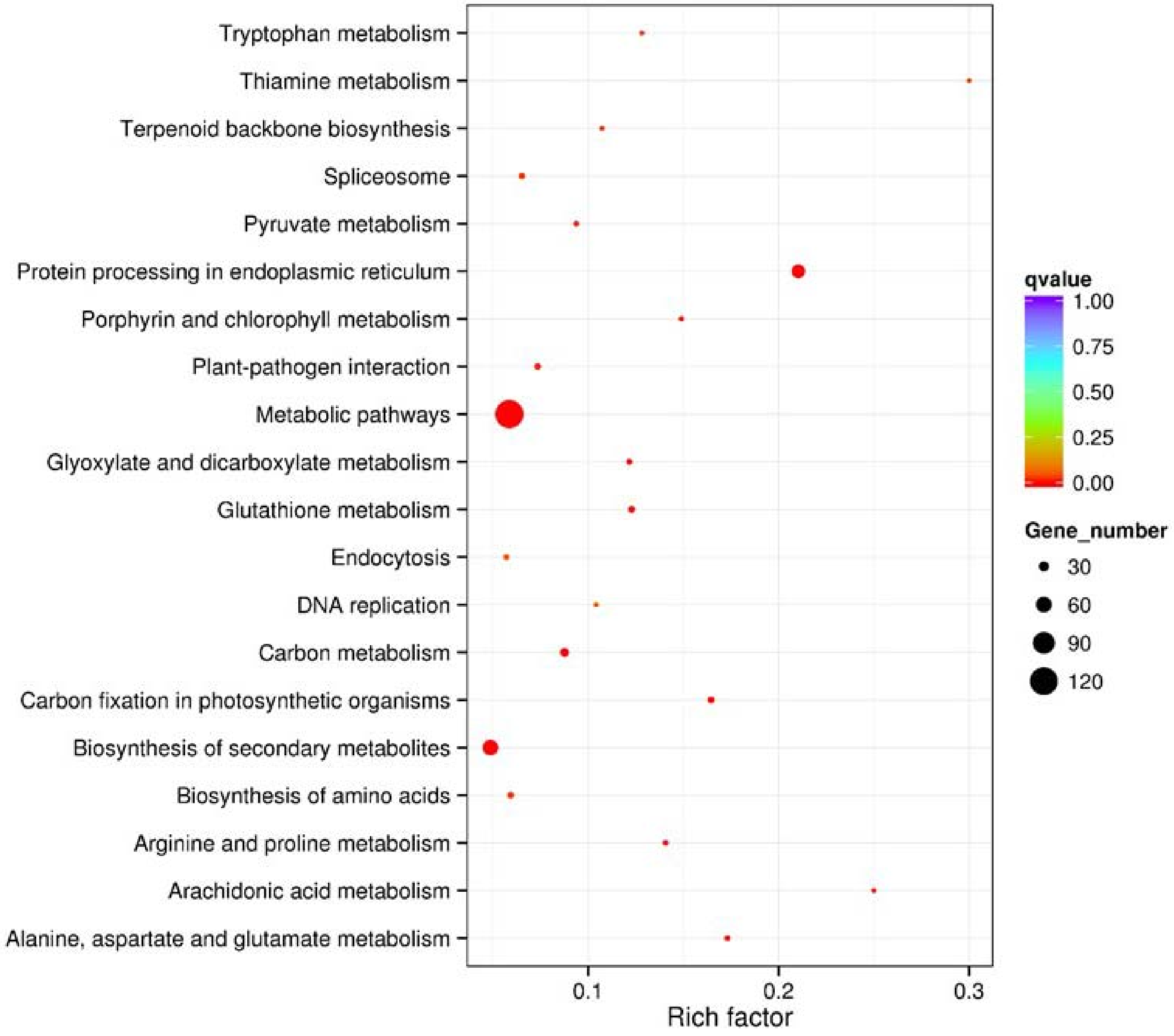

Supplementary Figure S5. The KEGG pathway enrichment analysis for the differentially expressed genes (DEGs) between PEP-deficient tobacco and wild-type plants.

The top 20 most significant enriched pathways (y-axis) were selected in KEGG scattered plot. The rich factor (x-axis) is the ratio of DEGs counts to the particular pathway over the annotated genes counts in the same pathway. The dot size represents the number of different genes and the color indicates the q-value (p value after normalization)
